# Dual targeting of PI3K and BCL‐2 overcomes ibrutinib resistance in aggressive mantle cell lymphoma

**DOI:** 10.1111/jcmm.17297

**Published:** 2022-03-30

**Authors:** Haige Ye, Shengjian Huang, Yang Liu, Zhihong Chen, Michael Wang, Vivian Changying Jiang

**Affiliations:** ^1^ Department of Lymphoma and Myeloma The University of Texas MD Anderson Cancer Center Houston Texas USA; ^2^ Department of Stem Cell Transplantation and Cellular Therapy The University of Texas MD Anderson Cancer Center Houston Texas USA

## Abstract

Despite significant efficacy of ibrutinib therapy in mantle cell lymphoma (MCL), about one‐third of MCL patients will display primary resistance. In time, secondary resistance occurs almost universally with an unlikely response to salvage chemotherapy afterwards. While intense efforts are being directed towards the characterization of resistance mechanisms, our focus is on identifying the signalling network rewiring that characterizes this ibrutinib resistant phenotype. Importantly, intrinsic genetic, epigenetic and tumour microenvironment‐initiated mechanisms have all been shown to influence the occurrence of the ibrutinib resistant phenotype. By using in vitro and in vivo models of primary and secondary ibrutinib resistance as well as post‐ibrutinib treatment clinical samples, we show that dual targeting of the BCL‐2 and PI3‐kinase signalling pathways results in synergistic anti‐tumour activity. Clinically relevant doses of venetoclax, a BCL‐2 inhibitor, in combination with duvelisib, a PI3Kδ/γ dual inhibitor, resulted in significant inhibition of these compensatory pathways and apoptosis induction. Our preclinical results suggest that the combination of venetoclax and duvelisib may be a therapeutic option for MCL patients who experienced ibrutinib failure and merits careful consideration for future clinical trial evaluation.

## 1 INTRODUCTION

Bruton's tyrosine kinase (BTK) inhibitors (BTKi) have been approved by the FDA for the treatment of relapsed/refractory (R/R) MCL; however, intrinsic and acquired BTKi resistance remains a clinically unmet challenge. Therefore, overcoming BTKi resistance is critical to improving clinical patient outcome. Targeting compensatory pathways that drive BTKi resistance by single agents or in combination could potentially overcome BTKi resistance. Duvelisib is a dual PI3Kδ/γ inhibitor that has shown modest preclinical activity in both ibrutinib‐sensitive and ibrutinib‐resistant (IR) MCL.^1^ Venetoclax is a specific BCL‐2 inhibitor that has been approved to treat relapsed chronic lymphocytic leukaemia (CLL)^2^ and shown clinical efficacy in MCL patients, especially when combined with ibrutinib.^3^, ^4^, ^5^ In IR MCL cells, sustained PI3K‐mTOR activation results in high expression of BCL‐2 and MCL‐1.^6^ Duvelisib treatment decreased BCL‐2 expression and sensitized venetoclax treatment in CLL.^7^ Therefore, we hypothesized that targeting PI3K by duvelisib and BCL‐2 by venetoclax would result in a synergistic anti‐MCL activity and overcome BTKi resistance. In this study, we investigated the in vitro and in vivo efficacy of duvelisib and venetoclax combination in IR MCL cells.

## 2 MATERIALS AND METHODS

### 2.1 Cell lines

Mantle cell lymphoma cell lines JeKo‐1, Maver‐1, Z138, JeKo‐R,^8^ JeKo BTK KD cells^8^ and JeKo‐Luc cells^8^ were cultured in RPMI 1640 medium supplemented with 10% FBS and 1% penicillin/streptomycin. HS‐5 cells were maintained in DMEM medium with 10% FBS and 1% penicillin/streptomycin.

### 2.2 Primary patient samples

Patient samples were obtained through a protocol approved by the Institutional Review Board at MD Anderson Cancer Center.

### 2.3 Reagents

Ibrutinib (S2680), venetoclax (S8048) and duvelisib (S7028) were purchased from Selleckchem.

### 2.4 Cell viability, cell apoptosis assay and Western blotting

These assays were performed as described previously.^8^

### 2.5 Reverse phase protein array analysis

JeKo BTK KD cells were treated with duvelisib and/or venetoclax for 24 h and subjected to reverse phase protein array (RPPA) analysis as described previously.^8^ Proteins with >twofold change between the combination and vehicle were selected to generate the heatmap.

### 2.6 Stroma‐mediated cell migration and viability assay

The bottom chamber of the Transwell system was pre‐seeded with 1 × 10^4^ HS‐5 and left to attach overnight. 1 × 10^5^ MCL cells were labelled with CMFDA, pre‐treated with duvelisib and/or venetoclax for 30 min and washed with PBS before adding to the upper chamber. Four hours later, CMFDA‐positive cells (bottom chamber migrated MCL cells) were expressed as a percentile of cells/well. JeKo‐1 cell viability in response to ibrutinib was tested in the presence and absence of HS‐5 cells using a similar procedure but without the Transwell system.

### 2.7 In vivo drug efficacy in mouse xenograft models

5 × 10^6^ JeKo‐Luc cells were injected subcutaneously into each 6–8‐week‐old NOD SCID IL2Rγ null (NSG) mice. Treatment was started 3 days following inoculation: vehicle, ibrutinib (50 mg/kg, oral, daily), venetoclax (50 mg/kg, oral, daily) and duvelisib (50 mg/kg, oral, daily) alone or in combination for 3 weeks. Tumours were measured weekly by IVIS Spectrum In Vivo Imaging System (Perkin Elmer).

### 2.8 Animal study approval

All experimental procedures and protocols were approved by the Institutional Animal Care and Use Committee of The University of Texas MD Anderson Cancer Center.

### 2.9 Statistics

Student's *t*‐test was used to calculate statistical significance.

## 3 RESULTS AND DISCUSSION

### 3.1 Duvelisib and venetoclax in combination synergistically inhibited the cell growth of ibrutinib‐resistant MCL models

**FIGURE 1:**
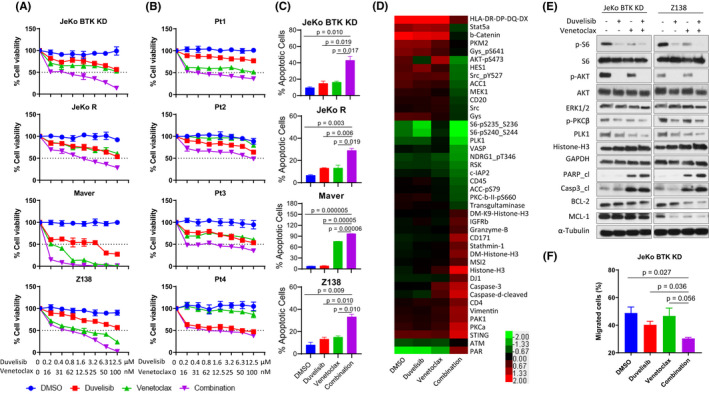
Duvelisib and venetoclax in combination synergistically reduced cell growth and induced apoptosis in MCL. (A, B) Four IR MCL cell lines (A) and four patient samples (B) were treated with DMSO, increasing doses of duvelisib (0–12.5 μM), venetoclax (0–100 nM) and the combination. Cell viability was detected at 72 h (A) or 24 h (B). (C) Four MCL IR cell lines were treated with vehicle, duvelisib (5 μM), venetoclax (100 nM for JeKo BTK KD and JeKo R, or 10 nM for Maver and Z138) and the combination. Cell apoptosis was determined by annexin‐V/PI staining, and both annexin‐V^+^/PI^−^ and annexin‐V^+^/PI^+^ subpopulations were considered apoptotic cells. Each treatment for cell viability and apoptosis was set up in triplicate and repeated at least two independent times. (D) 5 × 10^6^ JeKo BTK KD cells were treated with vehicle, 5 μM duvelisib, 100 nM venetoclax, or the combination for 24 h and harvested for RPPA analysis. Proteins with more than a twofold change between the combination and vehicle control were selected for heatmap generation using Cluster 3.0 and Java Treeview. Each treatment for RPPA was set up in triplicate. (E) Two MCL IR cell lines, JeKo BTK KD and Z‐138, followed the same treatments as RPPA analysis and protein lysates were collected for Western blotting. (F) CMFDA‐labelled JeKo BTK KD cells were pre‐treated for 30 min with either vehicle, 5 μM duvelisib or 100 nM venetoclax, alone and in combination before loading to the upper chamber of the Transwell migration system. The bottom chamber was pre‐seeded with CMFDA‐unlabelled HS‐5 monolayer overnight. At 4 h of incubation, the cell counts of CMFDA‐positive MCL cells migrated to the bottom chamber were determined by flow cytometry and total cell counts in the bottom. The percentage of CMFDA‐labelled MCL cells that migrated into the lower chamber out of total cells loaded into upper chamber were generated and plotted

All ibrutinib‐resistant cell lines and patient samples (except Pt4) were only slightly responsive to duvelisib as a single agent, whereas two cell lines (Z138 and Maver‐1) and two patient samples (Pt1 and Pt3) were sensitive to venetoclax as a single agent (Figure 1A,B). A striking synergy was observed in 3/4 cell lines and 3/4 patient samples, while a weaker combination effect was observed in Maver and Pt4 (Figure 1A‐C). This synergistic effect was also confirmed by isobologram analysis^9^ (Figure S1A‐B). Consistently, cell apoptosis (30.3%–97.0%) was induced by the combination therapy (combination index 0.36–0.77) in all tested cell lines (Figure 1C). The patient characteristics of the four patients used is summarized in Table S1.

To understand the underlying mechanism of action, we performed an unbiased proteomic analysis by reverse‐phase protein array (RPPA) analysis with 425 antibodies encompassing multiple signalling pathways (Figure 1D). PI3K/AKT/mTOR, PKC and PLK1 signalling pathways were predominantly downregulated in the combination‐treated cells, validating the intended targets. PI3K/AKT/mTOR plays a crucial role in IR,^6^ and blocking this pathway is a rational strategy for overcoming IR. PKC activation correlates with ibrutinib relapse in CLL.^10^ BTK activates and upregulates the expression of BCL‐2 family.^11^ PLK1, a central cell cycle regulator at G2/M phase, is associated with IR as previously shown.^12^ Moreover, the most upregulated proteins in the combination group were apoptosis‐related proteins such as caspases and Histone‐H3 (Figure 1D). These results were further validated by Western blotting (Figure 1E). Together, these data demonstrate that the combination of duvelisib and venetoclax overcomes IR synergistically through inhibition of the PI3K‐Akt‐mTOR pathway and activation of pro‐apoptotic signalling.

### 3.2 Duvelisib and venetoclax in combination inhibit significantly TME‐mediated cell migration

The tumour microenvironment (TME) plays a critical role in drug resistance in many cancer types.^13^ PI3K‐AKT signalling plays a crucial role in regulating the tumour‐TME interplay, and targeting this pathway *via* duvelisib has shown to disrupt the malignant B‐cell homing process.^14^, ^15^, ^16^ To investigate whether duvelisib and venetoclax in combination synergistically block directional cell migration, we performed a Transwell migration assay using IR JeKo BTK KD and human stroma cell line HS‐5 cell monolayer as the TME attractant (Figure S2A). A 30‐min pre‐treatment with the venetoclax and duvelisib combination significantly reduced cell migration towards the HS‐5 monolayer compared with controls (*p* = 0.027) and with venetoclax and duvelisib alone (*p* = 0.036 and *p* = 0.056, respectively; Figure 1F and Figure S2B). These data suggest that this combination has the potential to interfere with the chemoattractant release and, therefore, impacting directional migration.

### 3.3 The combination of duvelisib and venetoclax overcomes TME‐mediated ibrutinib‐resistance

**FIGURE 2:**
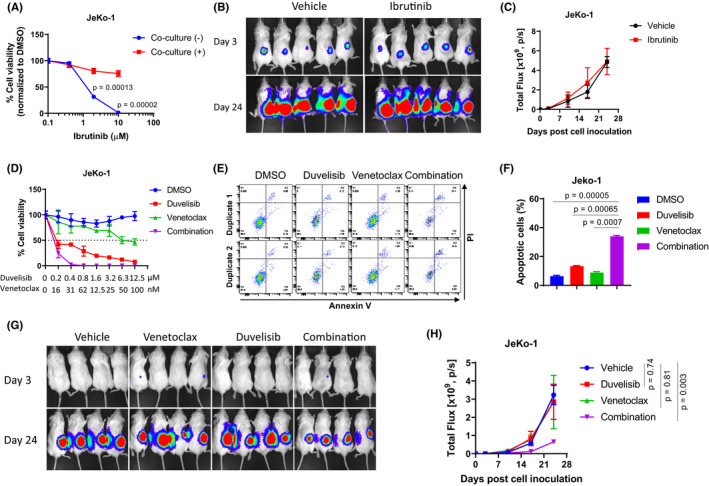
Duvelisib and venetoclax in combination circumvents TME‐mediated ibrutinib‐resistance in vitro and in vivo. (A) In vitro efficacy of ibrutinib in JeKo‐1 cells with or without co‐culture with HS‐5 cells at 72 h post‐treatment. (B, C) NSG mice were injected subcutaneously with 5 × 10^6^ JeKo‐Luc cells per mouse and treated orally once a day with vehicle or ibrutinib (50 mg/kg). Treatment was started on Day 3 following cell inoculation, and tumour burden was measured at Days 3, 17 and 24 post‐treatment by live imaging (B) and by luminescent flux (C). (D) 72‐h dose‐dependent cell viability assay performed on JeKo‐1 cells co‐cultured with HS‐5 cells. (E‐F) 24‐h cell apoptosis assay was conducted in JeKo‐1 cells co‐cultured with HS‐5 cells. The cells were treated with vehicle, 5 μM duvelisib, 100 nM venetoclax or the combination. Cell apoptosis was determined by annexin‐V/PI staining and flow cytometry, and both annexin‐V^+^/PI^−^ and annexin‐V^+^/PI^+^ subpopulations were calculated as apoptotic cells. (E) Representative flow cytometry data are shown for each treatment. (F) Plotted percentile values of apoptotic cells are shown for each treatment. Each cell viability and apoptosis assay were set up in triplicate and repeated at least two independent times. (G, H) 5 × 10^6^ JeKo‐Luc cells were injected into NSG mice subcutaneously and the tumour burden was measured by live imaging. Treatments were started 3 days after cell inoculation. Mice (*n* = 4) were treated orally, once a day, with vehicle, venetoclax (50 mg/kg), duvelisib (50 mg/kg), alone or in combination. (G) Luminescent images of live mice are shown for Day 3 and Day 24 post‐treatment. (H) Luminescent flux values plotted at days 3, 17 and 24 are shown for each treatment arm

To investigate the role of TME in tumour phenotypic changes, we co‐cultured ibrutinib‐sensitive JeKo‐1 cells with HS‐5 cells. JeKo‐1 cells became IR when co‐cultured with HS‐5 cells in vitro (*p* < 0.0001; Figure 2A). The effect of TME shifting the JeKo‐1 phenotype from sensitive to IR was also confirmed in vivo as mice bearing JeKo‐Luc‐derived xenografts showed no response to ibrutinib treatment (Figure 2B,C). Next, we determined whether duvelisib and venetoclax in combination could overcome TME‐associated IR. Similar to our primary IR models (JeKo BTK KD, Maver‐1 and Z138 cells) and the acquired IR model (JeKo R), venetoclax and duvelisib in combination overcame IR in the stroma/JeKo‐1 co‐culture (*p* = 0.0007; Figure 2D‐F). Furthermore, JeKo‐Luc‐derived xenograft models were resistant to single agent treatment but sensitive (*p* = 0.003) to the combination (Figure 2G,H). Altogether, these data demonstrate that this combination may potentially subvert primary, acquired and TME‐associated MCL IR in both, in vitro and in vivo.

Here, we demonstrated that duvelisib and venetoclax in combination overcomes IR‐MCL synergistically in vitro and in vivo. We investigated different types of IR including intrinsic, acquired and TME‐associated resistance mechanisms. The combination reduced tumour growth and migration in all IR cell types, indicating that this is a robust and sustained therapeutic strategy for overcoming IR. While the exact mechanism is still under investigation, we report here that PI3K‐AKT/mTOR and Bcl‐2 signalling are the main IR phenotype drivers. There are four PI3K inhibitors: idelalisib,^17^ duvelisib,^18^ alpelisib ^19^ and copanlisib ^20^ that have been approved by FDA. We tested three of the four PI3K inhibitors in MCL cells, excluding alpelisib (targeting PI3Kα) since PI3Kδ is highly expressed in haematologic malignancies,^21^ including MCL (Figure S3). Both duvelisib (targeting PI3Kδ/PI3Kγ) and copanlisib (pan‐class I PI3K inhibitor), but not idelalisib (targeting PI3Kδ), showed potent anti‐MCL activity (data not shown). MCL cells express all four isoforms of PI3K (Figure S3), so it is likely that targeting PI3Kδ only by idelalisib is not sufficient for inducing cytotoxicity in MCL. Expression of other isoforms in addition to PI3Kδ may compensate for the PI3K‐mediated tumour cell survival and growth. Our data on duvelisib and venetoclax in combination provides evidence to support the dual targeting of PI3K and BCL‐2 as a promising therapeutic strategy to overcome ibrutinib resistance in R/R MCL. It is likely copanlisib and venetoclax in combination will also show anti‐MCL synergy.

## CONFLICT OF INTEREST

M.W. is a consultant for the following: InnoCare, Loxo Oncology, Juno, Oncternal, CStone, AstraZeneca, Janssen, VelosBio, Pharmacyclics, Genentech and Bayer Healthcare. His research is funded by the following: Pharmacyclics, Janssen, AstraZeneca, Celgene, Loxo Oncology, Kite Pharma, Juno, BioInvent, VelosBio, Acerta Pharma, Oncternal, Verastem, Molecular Templates, Lilly and Innocare. All other authors declare no competing financial interests.

## AUTHOR CONTRIBUTIONS

**Haige Ye:** Data curation (equal); Formal analysis (equal); Investigation (lead); Methodology (equal); Writing – review & editing (supporting). **Shengijian Huang:** Data curation (equal); Formal analysis (equal); Investigation (supporting); Methodology (equal); Writing – original draft (lead); Writing – review & editing (supporting). **Yang Liu:** Formal analysis (supporting); Investigation (supporting). **Zhihong Chen:** Methodology (equal). **Michael Wang:** Conceptualization (lead); Funding acquisition (lead); Resources (lead); Supervision (supporting); Writing – review & editing (supporting). **Vivian Changying Jiang:** Conceptualization (supporting); Data curation (supporting); Formal analysis (supporting); Investigation (supporting); Methodology (supporting); Project administration (lead); Supervision (lead); Writing – original draft (supporting); Writing – review & editing (lead).

## Supporting information

Supplementary Material
