# Supplementary material for: Dual targeting of PI3K and BCL‐2 overcomes ibrutinib resistance in aggressive mantle cell lymphoma
Source: J Cell Mol Med. 2022 Mar 30;26(10):3068–73. doi: 10.1111/jcmm.17297 (PMC9097828; doi:10.1111/jcmm.17297)
Supplement: Supplementary file 1 — Supplementary Material [file JCMM-26-3068-s001.pdf]

Supplementary figures and legends

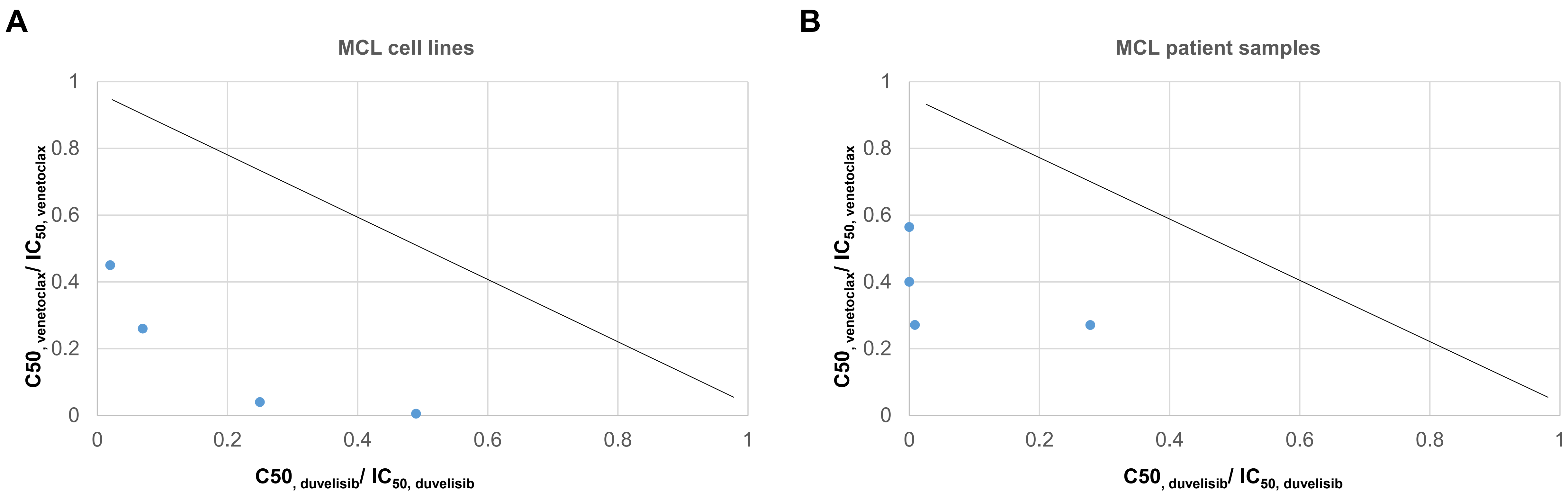

Supplementary Figure S1. Isobologram analysis for combinational effect of duvelisib and venetoclax in MCL cell lines (n = 4) (A) and primary patient samples (n = 4) (B).

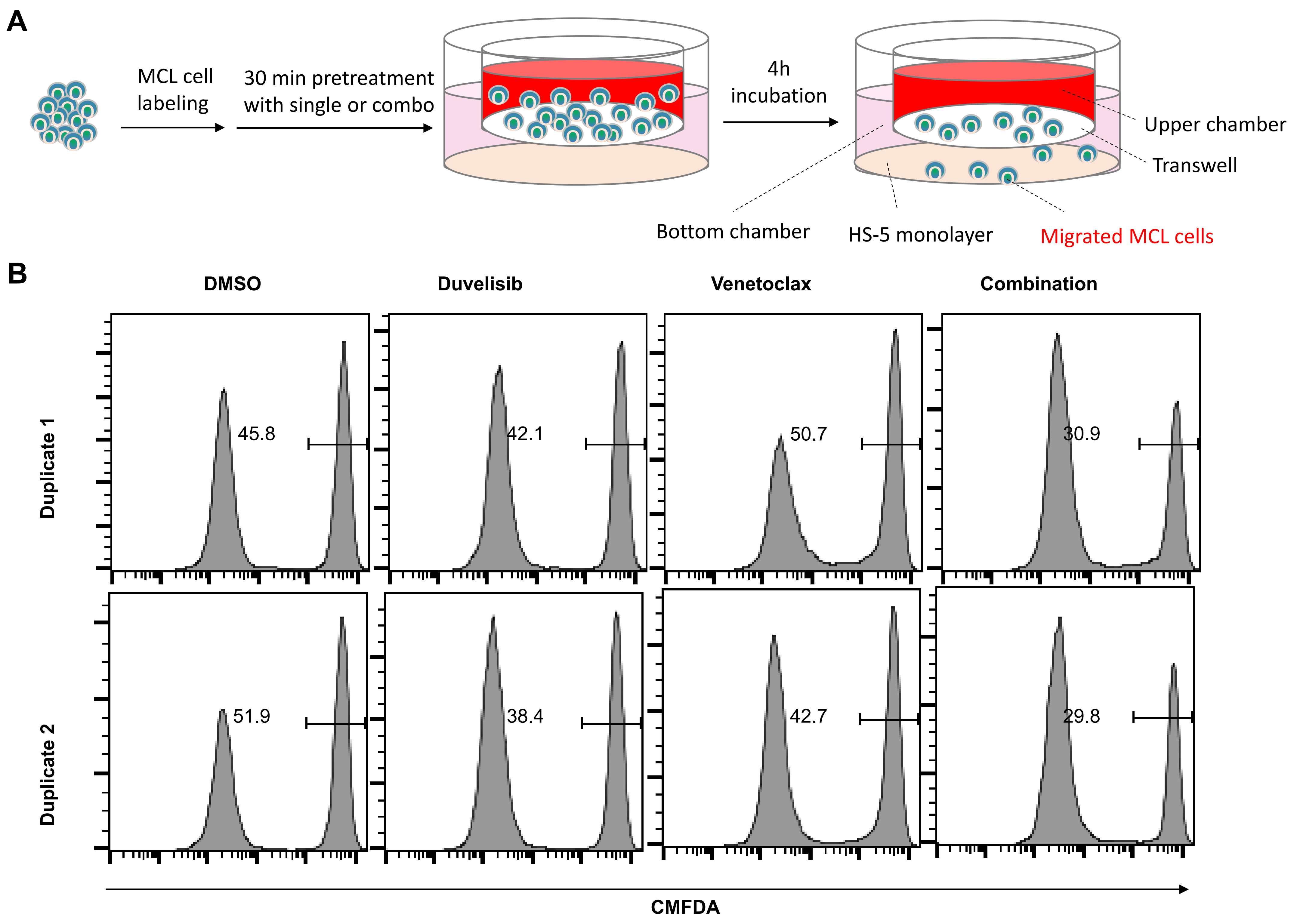

Supplementary Figure 2: Duvelisib and venetoclax combination showed greater inhibition in TME-mediated cell migration *in vitro*. (**A**) Schematic design for *in vitro* MCL cell migration. Ibrutinib-resistant JeKo BTK KD cells were stained with CellTracker™ Green CMFDA Dye and pretreated with DMSO, duvelisib at 5 µM, venetoclax at 100 nM, alone or in combination, for 30 minutes and washed with 1x PBS before adding to the upper chamber for the trans-well migration assay. The bottom chamber was pre-seeded with human stroma cell lines HS-5 overnight. The trans-well systems were incubated for 4 hours before collection of migrated MCL cells and flow cytometry analysis. (**B**) Migrated MCL cells were detected by flow cytometry based on CMFDA-labeling. Representative flow cytometry data are shown. The CMFDA-positive cells (the right peak) represent the migrated MCL cells into the lower chamber that were CMFDA-labelled before loading into the upper chamber, and the negative cells (the left peak) represent the HS-5 cells which were not labeled with CMFDA.

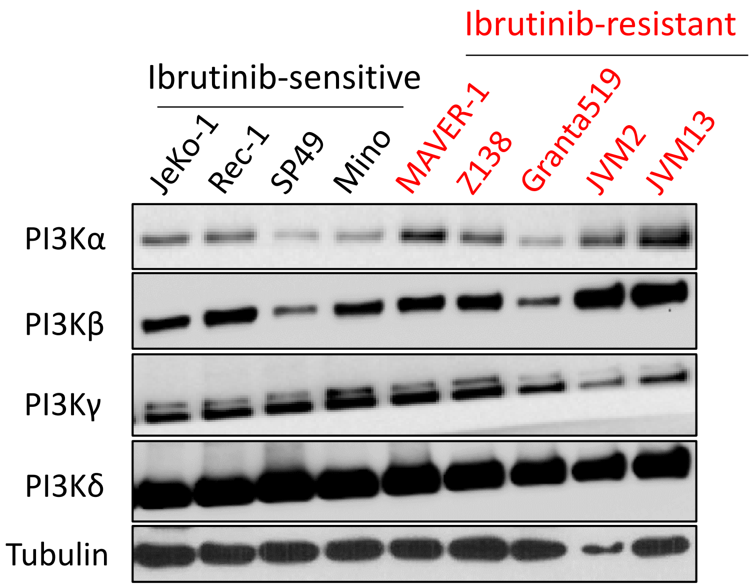

Supplementary Figure S3: Expression of PI3K isoforms in ibrutinib-sensitive (black) and –resistant (red) MCL cell lines.

Supplementary Table S1. Clinical characteristics of patients with MCL.

Characteristics	Patient 1	Patient 2	Patient 3	Patient 4
**Sex**	M	M	F	M
**Age**	75	69	70	50
**Bone marrow involvement**	Positive	Negative	Positive	low
**Peripheral blood involvement**	Positive	Positive	Positive	Positive
**Karyotype**	Complex	Normal	Complex	Normal
**TP53**	NA	Positive	NA	NA
**Ki67**	20%	>95%	65%	40-50%
**Sox11**	NA	Positive	Positive	NA
**Histology type**	Blastoid	Blastoid	Blastoid	classic
**Prior therapy**	1	1	1	2
**BTKi therapy**	Acalabrutinib	Ibrutinib	Ibrutinib	Ibrutinib
**Response to BTKi therapy**	Relapsed	Relapsed	Relapsed	Relapsed
**Ibrutinib-resistant**	Yes	Yes	Yes	Yes
